# Single-chain human Follicle-stimulating hormone promotes ovarian and testicular growth and improves pregnancy performance in mice

**DOI:** 10.1371/journal.pone.0349398

**Published:** 2026-05-14

**Authors:** Thi Tho Nguyen, Van Hai Nong, Thi Mong Diep Nguyen

**Affiliations:** 1 Quy Nhon University, Gia Lai, Vietnam; 2 Graduate University of Science and Technology, Vietnam Academy of Science and Technology, Hanoi, Vietnam; 3 Institute of Biology, Vietnam Academy of Science and Technology, Hanoi, Vietnam; King Saud University / Zagazig University, EGYPT

## Abstract

Follicle-stimulating hormone (FSH) is a central regulator of folliculogenesis and reproductive function. To enhance its stability and pharmacological activity, we designed a single-chain (sc) gonadotropin containing two (LNN) specific N-glycosylation sites (Asn-X-Ser/Thr) in the linker, fusing the two subunits polypeptide chain sequences. This study evaluated the *in vivo* bioactivity of sc-hFSH LNN compared with the Standard hFSH NIBSC (hFSH NIBSC) reference preparation in ICR mice (an outbred Swiss strain that has been established and maintained in Vietnam). A total of 285 animals (21-day-old) were randomly assigned to three groups: (1) sc-hFSH LNN, (2) hFSH NIBSC, and (3) Control group receiving saline only. For each experimental condition, five animals per group were used. Depending on the experimental design, different concentrations (0.2, 1, 2.5, and 5 µg/mL) were administered. Experiment 1 tested a low-dose regimen, consisting of injecting five times a fifth of the total FSH dose (5 x 0.04 and 5 x 0.2 µg/mL) at 12-hour intervals. Experiment 2 used a single-dose injection (0.2 and 1 µg/mL). The control group received saline injections only. Mice were sacrificed 72 hours after treatment. Ovaries and uteri were collected and compared between the groups. Subsequent experiments were conducted using a single-dose injection protocol in 21-day-old female and male mice. Ovaries, uteri, testes, vas deferens, and blood samples were collected and compared between the groups. The mating experiment was performed in adult mice (8-week-old) with 24 males and 24 females. The results showed that a single-injection regimen produced a more potent stimulation than multiple split doses, and that a nonlinear dose-response was observed, with significant increases in ovarian and testicular weights measured only at 5 µg/mL compared to 1 µg/mL. In adult females, pre-mating treatment with sc-hFSH LNN and hFSH NIBSC resulted in the highest pregnancy rates and largest litter sizes compared to control, suggesting a superior ability to support follicular maturation and ovulation *in vivo*. These findings show that the engineered sc-hFSH LNN is biologically active, has the potential to improve reproductive outcomes, and may serve as a promising alternative to conventional heterologous FSH in both reproductive biology research and future biopharmaceutical applications.

## Introduction

Follicle-stimulating hormone (FSH) is a glycoprotein gonadotropin secreted by the anterior pituitary in response to hypothalamic gonadotropin-releasing hormone (GnRH). Along with luteinizing hormone (LH), it plays a central role in regulating gonadal function by binding to specific membrane receptors in the ovaries and testes, thereby promoting steroidogenesis and gametogenesis. Structurally, FSH is a heterodimeric glycoprotein composed of a common α-subunit and a hormone-specific β-subunit, both of which are N-glycosylated: αN52 and αN78 in the α-subunit, and βN7 and βN24 in the β-subunit. These glycans contribute critically to hormone activity, with αN52 implicated in stabilizing the receptor–FSH dimer complex and activating G-protein signaling, while β-linked glycans largely determine *in vivo* half-life and overall bioactivity [1].

Functionally, FSH exerts sex-specific roles. In males, it regulates spermatogenesis through Sertoli cells, activating MAPK, AMPK, TGF-β/Smad, PKA/cAMP, and PI3K/PKB pathways to enhance nutrient uptake, protein synthesis, and SC differentiation, processes essential for germ cells development and the initiation of spermatogenesis [2–5]. In females, FSH is indispensable for folliculogenesis, stimulating aromatase activity in granulosa cells, enhancing estrogen biosynthesis, and inducing LH receptor expression to ensure ovulation and corpus luteum formation during the mid-cycle LH surge [6–8].

FSH is widely applied in assisted reproductive technologies (ART), particularly for controlled ovarian stimulation. Early urinary-derived preparations (uFSH) have been replaced by recombinant FSH (rFSH) due to their superior purity, consistency, and bioactivity. Recently, novel FSH analogs with structural modifications have been developed to improve stability and pharmacodynamics [8–10]. In our most recent study, the single-chain hFSH with an N-glycosylated linker (sc-hFSH LNN) represents a promising innovation: sc-hFSH LNN expressed in HEK293 and CHO K1 cells demonstrated robust *in vitro* activity, significantly stimulating cAMP and progesterone production in mLTC cells expressing the human FSH receptor. *In vivo*, sc-hFSH LNN markedly increased ovarian weight and serum estradiol levels, up to eightfold higher than native hFSH (hFSH SIAFP from NHPP, Torrance, CA, USA) [11].

Despite FSH’s pivotal roles in reproductive biology, most clinical applications remain focused on female infertility. Data on its potential benefits in males are limited, though recombinant FSH has been shown experimentally to increase testicular weight, seminiferous tubule dimensions, and the number of Sertoli and germ cells, confirming its role in sustaining spermatogenesis [12–15]. Currently, FSH therapy is indicated mainly for hypogonadotropic hypogonadism, while its utility in idiopathic male infertility remains under investigation.

To expand understanding of sc-hFSH LNN’s therapeutic potential, we further evaluated its *in vivo* bioactivity in both prepubertal male and female ICR inbred mice. This model is ideal due to its intact but quiescent hypothalamic-pituitary-gonadal axis and sensitivity to exogenous gonadotropin hormones [16]. To obtain further evidence regarding the role of sc-hFSH LNN in a mammalian model, we assessed ovarian and uterine growth in prepubertal females, testicular parameters in prepubertal males, and, importantly, the pregnancy rate in mature female mice that mated with mature male mice was also determined. This study provides new insights into the reproductive benefits of engineered single-chain FSH analogs, highlighting their potential as stable and effective alternatives to conventional gonadotropins.

## Methods

### Experimental animals

ICR mice (Swiss breed) were provided by the Stem Cell Institute, University of Science, Vietnam National University, Ho Chi Minh City, and were maintained under controlled conditions (temperature: 26 ± 2 °C, humidity: 55 ± 10%, and a 12-hour light/dark cycle). The health status of the animals was monitored by observing their physical activity and feeding behavior. Animals were fed standard laboratory chow (Ho Chi Minh City, Vietnam) *ad libitum* and had free access to drinking water.

All mouse studies were conducted at the Biotechnology Center (Ho Chi Minh City, Vietnam) in accordance with protocols approved by the Local Animal Care and Use Committee. Procedures were designed to minimize animal suffering, and all experimental animals were humanely euthanized by exposure to gradually increasing concentrations of CO₂, following the recommendations of Directive 2010/63/EU.

### Ethics statement

This research was conducted in compliance with high ethical standards concerning animal welfare. All experimental procedures were carried out in accordance with the UK Animals (Scientific Procedures) Act, 1986, associated guidelines, and the EU Directive 2010/63/EU for animal experiments, and were approved by the Local Animal Care and Use Committee (Approval No. SD-CN2/024 20/05).

### Chemicals

All chemicals were purchased from Sigma-Aldrich unless otherwise stated. The hFSH-NIBSC hormone standard (WHO International Standard, code: 92/510) was obtained from the UK, and hCG was supplied by the French National Institute for Agriculture, Food and Environment (INRAe). The sc-hFSH LNN protein was produced from the CHO K1 cell line [11].

### *In vivo* bioassays

- **Uterus and ovaries collection**

**Fig 1:**
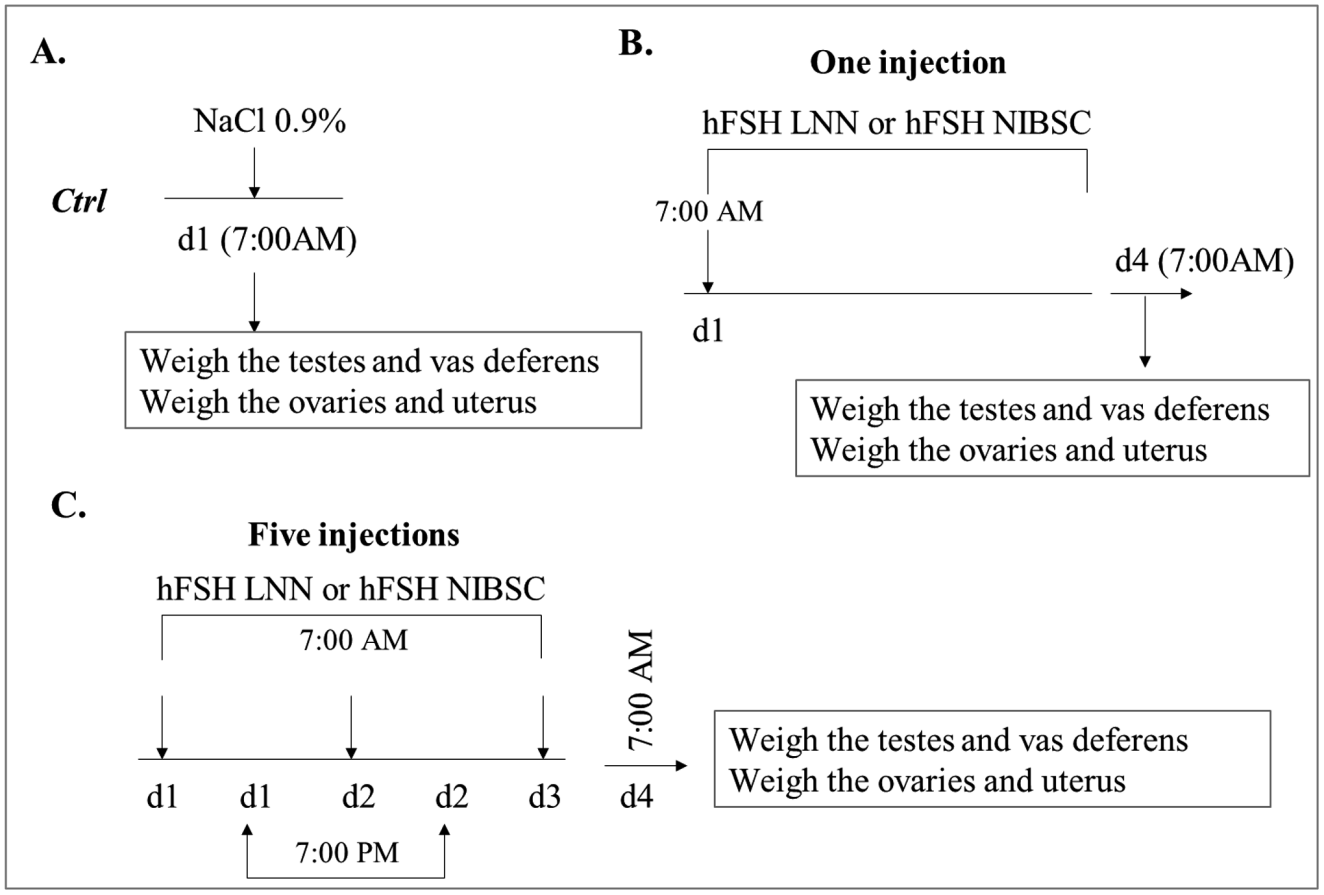
Experimental design of single- and five-dose regimen in male and female mice.

Female ICR mice (21-day-old) were divided into three groups: (1) sc-hFSH LNN, (2) hFSH NIBSC, and (3) a control group receiving saline only, and each experiment was performed in three independent replicates. These *in vivo* assays are based on: **1)** the one described by Cole and Erway consisting of a single injection 0.2 and 1 µg/100µl/animal at t = 0 [17], and **2)** the European Pharmacopoeia (EP7.0) recommendations with the same sample doses divided into five separate injections of 0.04 and 0.2 µg/100µl/animal at t = 0 h, 12 h, 24 h, 36 h, and 48 h, as shown in Fig 1 **A, B, C** [18]. All animals were sacrificed at time t = 72 hours. The two assays were performed using lots of 5 animals for each treatment.

Each animal received either a single or five subcutaneous injections of sc-hFSH LNN or hFSH NIBSC dissolved in saline, supplemented with 5 IU hCG for females, or 100 µL of saline alone as Control. Treatments were administered to 21-day-old animals, 72 hours prior to sacrifice by CO_2_ gas exposure at gradually increasing concentrations, as recommended by Directive 2010/63/EU. A midline incision was then carefully performed, and the abdominal contents were examined.

**Fig 2:**
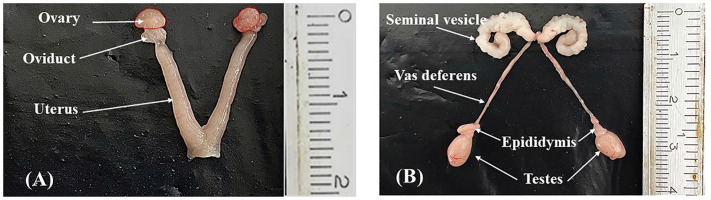
Morphology of reproductive organs from female mice (A) and male mice (B).

The uterus and ovaries were excised and carefully dissected to remove fat, oviducts, vagina, and all adherent membranes. When present, uterine fluid was removed by puncture, and the uterus and ovaries were blotted before measuring dry organ weights (Fig 2). All weights were determined using a Shimadzu AUW-220D balance (Shimadzu, Japan) with a precision of 0.1 mg.

- **Testes, Vas deferens, Ovaries, Uterus and blood collectio** **n**

Each 21-day-old animal (105 males and 105 females for three groups) received a single injection of 1, 2.5, or 5 µg/100µl/animal of either sc-hFSH LNN or hFSH NIBSC dissolved in saline, and supplemented with 5 IU hCG for females, or 100 µL of saline alone as Control at t = 0 (Fig 1 **A, B**). They were then sacrificed 72 hours later by CO_2_ gas exposure at gradually increasing concentrations, as recommended by Directive 2010/63/EU, followed by rapid cardiac blood collection. A midline incision was then carefully performed, and the abdominal contents were examined.

For females, the uterus and ovaries were excised and carefully dissected to remove fat, oviducts, vagina, and all adherent membranes. When present, uterine fluid was removed by puncture, and the uterus and ovaries were blotted before measuring dry organ weights (Fig 2A). For males, the testes and vas deferens were collected, with the epididymides and fat removed. When present, vas deferens fluid was removed by puncture, and both testes and vas deferens were blotted before measuring dry organ weights (Fig 2B). All weights were determined using a Shimadzu AUW-220D balance (Shimadzu, Japan) with a precision of 0.1 mg. Three independent experiments were performed for each group.

- **Enzyme-linked immunosorbent assay (ELISA)**

Blood samples were centrifuged at 2000 rpm for 10 min, and the serum was then collected and stored at −80°C until further analysis. Serum was collected for ELISA analysis to determine the concentrations of estradiol (E2), progesterone (P4), testosterone (T), and follicle-stimulating hormone (FSH). The ELISA kits used in this study were specific for E2 (Thermo Fisher; #ELR013), P4 (Thermo Fisher; #EELR011), T (Thermo Fisher; #EELR009), and FSH (Thermo Fisher; #EEL097). Each sample was tested in duplicate in at least three independent experiments.

### Determination of the Reproductive Index

Adult female and male ICR mice (8 weeks old) with previously confirmed fertility were randomly assigned to treatment groups according to the following experimental design:

**Assay 1**

Group 1 (n = 3 females): Females were injected with saline solution only.

Group 2 (n = 3 females): Females were injected with 5 µg/mL hFSH NIBSC per mouse.

Group 3 (n = 3 females): Females were injected with 5 µg/mL sc-hFSH LNN per mouse.

Forty-eight hours later, all female mice were injected with 10 IU hCG to induce ovulation. The females were then housed overnight with fertile male mice at a 1:1 ratio. This experiment was independently repeated twice.

**Assay 2**

Group 1 (n = 5 males and 5 females): Both male and female mice were injected with saline solution.

Group 2 (n = 5 males and 5 females): Female mice were injected with 5 µg/mL hFSH NIBSC per mouse, while male mice received saline only.

Group 3 (n = 5 males and 5 females): Both male and female mice were injected with 5 µg/mL sc-hFSH LNN per mouse.

During treatment, male and female mice were housed separately. Forty-eight hours later, all female mice were injected with 10 IU hCG to induce ovulation. Females were then paired overnight with males at a 1:1 ratio for mating.

The following morning, females were examined for the presence of a vaginal plug, separated from the males, and monitored for signs of pregnancy. Pregnant females were housed individually. From day 18 to day 21 after mating, cages were checked each morning for the presence of pups, and litter size was recorded.

The reproductive index was calculated as follows:


$$\begin{array}{c} \text{Reproductive index }(\%)=(\text{Number of pregnant females}/\text{Number of mated females})\times \text{ 100} \end{array}$$

### Statistical analyses

The GraphPad 5 package (GraphPad Software, San Diego CA) was used for the statistical evaluation. Each experiment above was repeated three times. Data are presented as mean ± standard deviation (SD). Statistical analyses were done using one-way ANOVA followed by the Dunnett’s test posttest. For all statistical analyses, P < 0.05 was considered significant.

## Results

### Effect of single-chain hFSH LNN on Uterus and Ovaries in immature female mice (21-day-old)

We evaluated the activity of sc-hFSH LNN in 21-day-old female mice, with hFSH NIBSC used as a positive control. The sc-hFSH LNN and hFSH NIBSC were tested under two dosing regimens: (1) a single subcutaneous injection, 72 h before sacrifice (t = 0), or (2) the same total dose equally divided into five injections given at t = 0 h, 12 h, 24 h, 36 h and 48 h, with sacrifice at t = 72 h.

**Fig 3:**
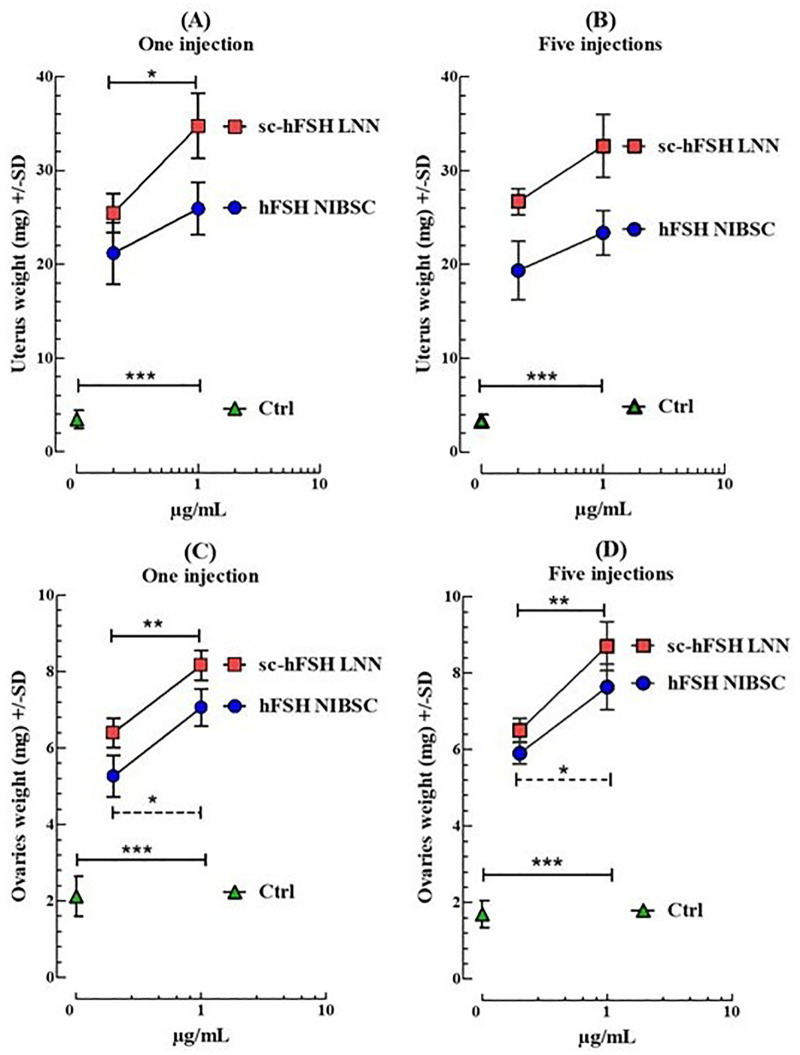
*In vivo* FSH bioactivity of single-chain hFSH hormone in immature female mice (21-day-old). The immature female mice (21-day-old) received a unique subcutaneous injection of one of these doses: 0 µg (Δ, green, only physiological saline as Ctrl), 0.2 µg/mL or 1 µg/mL of hFSH NIBSC (○, blue); and 0.2 µg/mL or 1 µg/mL of sc-hFSH LNN (□, red); or five subcutaneous injections of doses of 0 µg (Δ, green, only physiological saline as Ctrl), 0.04 µg/mL or 0.2 µg/mL of hFSH NIBSC (○, blue); and 0.04 µg/mL or 0.2 µg/mL of sc-hFSH LNN (□, red). Uterus and ovaries weight were determined 72 hours post injection. Each group had five animals per experiment. The experiment was performed independently three times. GraphPad represents Mean±SD, n = 3. Change in uterus weight (A) and ovaries weight (C) in female mice after a single injection and (C, D) for five injections. * p < 0.05, ** p < 0.01, *** p < 0.001, one-way ANOVA; Dunnett’s multiple comparisons test; different letters indicate statistically significant differences.

The results in Fig 3 show that both sc-hFSH LNN and hFSH NIBSC significantly increased ovarian and uterine weights compared to the control group (treated with saline only), with comparable responses at the same concentration (µg/mL). However, at the highest concentration (5 µg/mL), the single-injection regimen produced a stronger stimulatory effect than the five-injection regimen (Fig 3A). We then evaluated the effect of a single subcutaneous injection of higher doses of the sc-hFSH LNN, with the expectation of observing a dose-dependent increase in ovarian and uterine weights.

**Fig 4:**
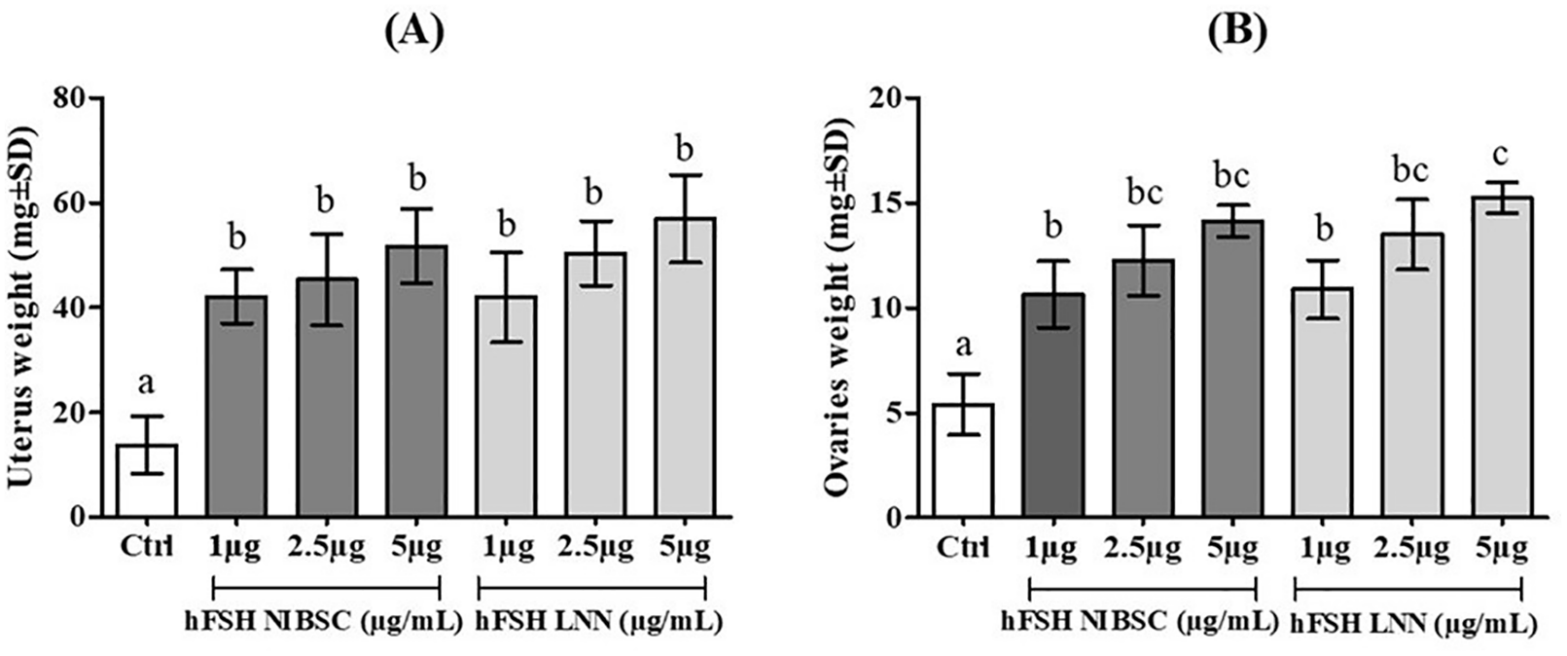
Effect of single chain hFSH LNN on uterus and ovaries of immature female mice (21-day-old) with single subcutaneous injection. Ctrl (Control, treatment with only physiological saline). Change in uterus weight **(A)** and ovaries weight **(B)** in immature female mice (21-day-old) after a single injection of sc-hFSH LNN or hFSH NIBSC at three different doses: 1, 2.5 and 5 µg/mL. Each group had five animals per experiment. The experiment was performed independently three times. GraphPad represents Mean±SD, n = 3. p < 0.05, one-way ANOVA; Dunnett’s multiple comparisons test; different letters indicate statistically significant differences.

**Fig 5:**
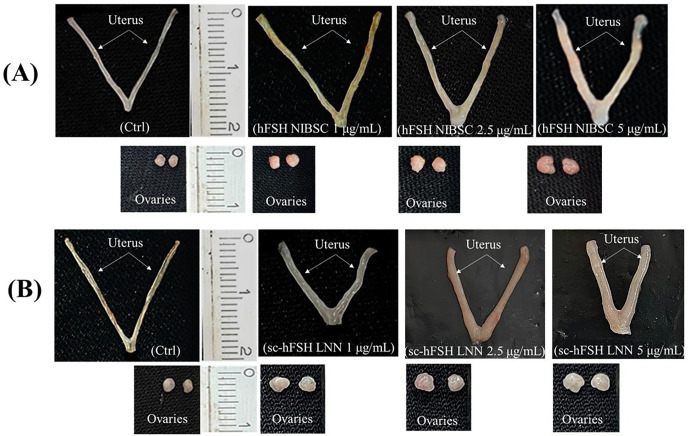
Morphology of reproductive organs from immature female mice (21-day-old) treated with hFSH NIBSC (A) and sc-hFSH LNN(B).Ctrl (Control, treatment with only physiological saline).

However, as shown in Figs 4 and 5, there was no significant difference in ovarian or uterine weight gain across most tested concentrations of sc-hFSH LNN. Only at the 5 µg/mL dose was a statistically significant increase in ovarian weight observed compared to the 1 µg/mL dose. Similarly, the hFSH NIBSC preparation showed no significant differences in ovarian or uterine responses at any tested concentration.

### Effect of single-chain hFSH LNN on the serum FSH, Estradiol and Progesterone levels in immature female mice (21-day-old)

**Fig 6:**
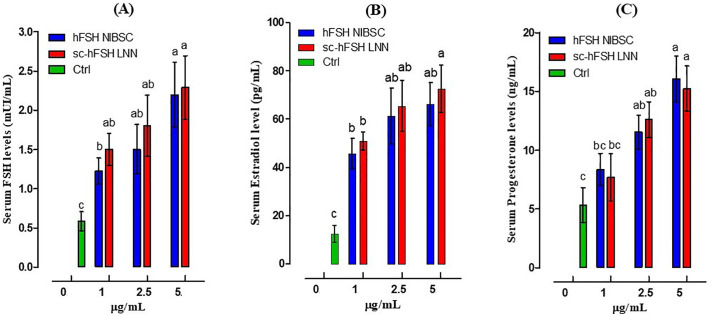
Effect of single-chain hFSH LNN on the serum FSH, Estradiol and Progesterone levels in immature female mice (21-day-old). **(A)** Serum FSH level, **(B)** Serum estradiol level, **(C)** Serum progesterone level. Each group had five animals per experiment. The experiment was performed independently three times. GraphPad represents Mean±SD, n = 3. p < 0.05, one-way ANOVA; Dunnett’s multiple comparisons test; different letters indicate statistically significant differences.

The efficacy of sc-hFSH LNN was further evaluated by measuring serum FSH, estradiol, and progesterone concentrations in 21-day-old female mice at 72 hours after sc-hFSH LNN injection. As shown in Fig 6, treatment with sc-hFSH LNN for 72 hours significantly increased serum FSH, estradiol, and progesterone levels compared with the control. Similar results were observed in the group treated with hFSH NIBSC. Among the tested concentrations, the 5 µg/mL sc-hFSH exhibited the greatest effect.

### Effects of single-chain hFSH LNN on testes and vas deferens of immature male mice (21-day-old)

21-day-old male mice were injected subcutaneously with sc-hFSH LNN and hFSH NIBSC, and 72 hours after injection, the testes and vas deferens were collected and weighed from five animals per dose.

**Fig 7:**
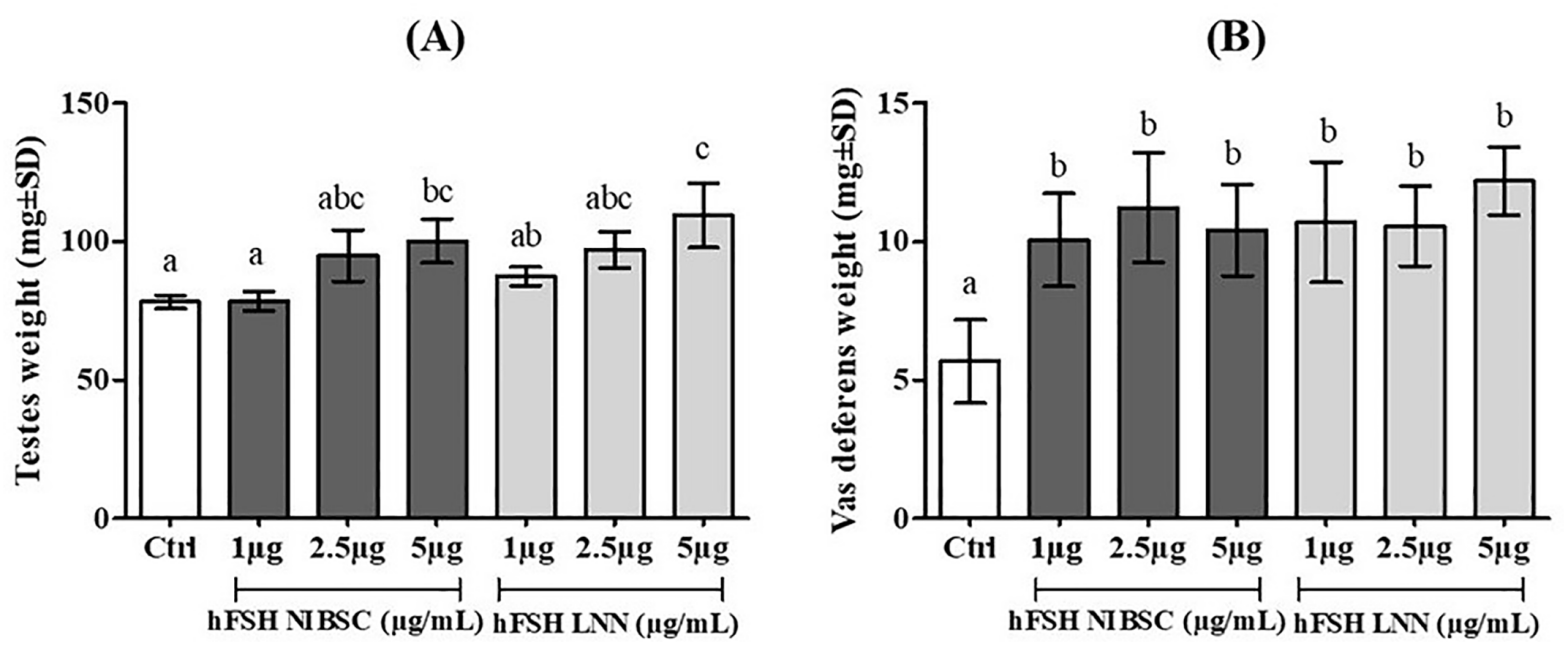
Effect of single chain hFSH LNN on weight of testes and vas deferens of immature male mice (21-day-old) with single subcutaneous injection. Ctrl (Control, treatment with only physiological saline). Change in testes weight **(A)** and vas deferens weight **(B)** in immature male mice (21-day-old) after a single injection of sc-hFSH LNN or hFSH NIBSC at three different doses: 1, 2.5 and 5 µg/mL. Each group had five animals per experiment. The experiment was performed independently three times. GraphPad represents Mean±SD, n = 3. p < 0.05, one-way ANOVA; Dunnett’s multiple comparisons test; different letters indicate statistically significant differences.

**Fig 8:**
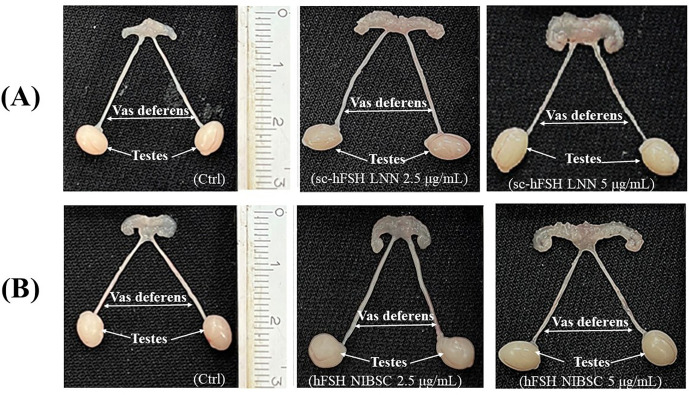
Morphology of reproductive organs from immature male mice (21-day-old) treated with hFSH NIBSC (A) and sc-hFSH LNN(B).Ctrl (Control, treatment with only physiological saline).

Figs 7 and 8 results show that the weight gain response of the seminiferous tubules occurred for all 3 doses tested with sc-hFSH LNN. However, for the testes, there was only a significant effect at the highest dose of 5 µg/mL compared to the control. Similar results were obtained for hFSH NIBSC.

### Effect of single-chain hFSH LNN on the serum FSH and Testosterone levels in immature male mice (21-day-old)

**Fig 9:**
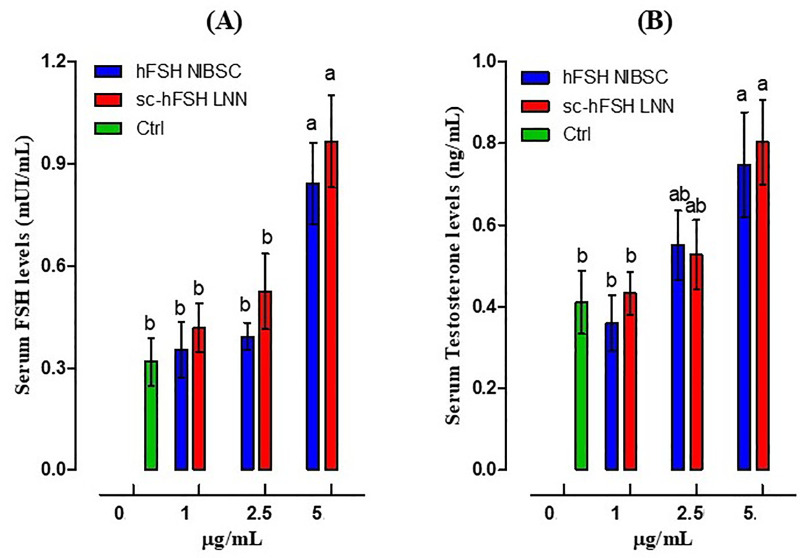
Effect of single-chain hFSH LNN on the serum FSH and Testosterone levels in immature male mice (21-day-old). **(A)** Serum FSH level, **(B)** Serum testosterone level. Each group had five animals per experiment. The experiment was performed independently three times. GraphPad represents Mean±SD, n = 3. p < 0.05, one-way ANOVA; Dunnett’s multiple comparisons test; different letters indicate statistically significant differences.

Compared with the saline-injected control, a single sc-hFSH LNN injection significantly increased serum FSH and testosterone concentrations, with the maximal effect observed at 5 µg/mL. No significant differences were detected at the 1 and 2.5 µg/mL doses compared with the control (p > 0.05; Fig 9). A similar result was also found in the group treated with hFSH NIBSC.

### Effects of single-chain hFSH on fertility rates in mice

When adult ICR female mice were randomly assigned to receive hFSH NIBSC, sc-hFSH LNN, or Ctrl (saline solution), and then mated with untreated adult male mice, the results showed that the number of mice that became pregnant and delivered pups was 55.56% for hFSH NIBSC (5/9), 66.67% for hFSH LNN (6/9), and 33.33% for Ctrl (3/9) (Table 1, Assay 1).

**Table 1:** Pregnancy rate of adult mice (8 weeks old) after sc-hFSH LNN injection.

Treatment	Number of paired mice	Number of pregnant mice	Fertility rate (%)	Number of pups at weaning, 21-day-old (mean±SEM)
**Assay 1:** Adult female mice treated with hormones, adult male mice not treated with hormones (n = 3, 3 animals/group)				
**Ctrl** (physiological saline)	9	3	33.33 ± 0.00^b^	**26** (7.67 ± 0.33^b^)
**hFSH NIBSC** (5 µg/mL)	9	5	55.56 ± 11.11^b^	**51** (16.67 ± 2.33^a^)
**hFSH LNN** (5 µg/mL)	9	6	66.67 ± 0.00^a^	**60** (20.00 ± 0.58^a^)
**Assay 2:** Adult female and male mice treated with hormones (n = 1, 5 animals/group)				
**Ctrl** (physiological saline)	5	1	20	7
**hFSH NIBSC** (5 µg/mL)	5	3	60	26
**hFSH LNN** (5 µg/mL)	5	3	60	32

The experiment was performed independently three times for Assay 1. p < 0.05, one-way ANOVA; Dunnett’s multiple comparisons test; different letters in the same column indicate statistically significant differences.

In addition, single-chain hormones showed positive effects when administered to immature male mice. We treated both adult male and female mice (8 weeks old) with the hormones and mated them 72 hours after injection at a 1:1 ratio. Preliminary results indicated that sc-hFSH LNN significantly increased the pregnancy rate and litter size compared with the control group. Treatment with the hFSH NIBSC hormone produced similar effects; however, the number of pups was lower than in the group treated with the sc-hFSH LNN (Table 1, Assay 2).

### Discussion

This study provides new evidences into the biological activity of a novel single-chain form of human FSH (sc-hFSH LNN), in which the α and β subunits are covalently linked through a peptide linker containing two N-glycans, complementing our recent findings on its prolonged pharmacological action due to an extended half-life [11]. The unique structural design of sc-hFSH LNN aims to enhance stability, prolong serum half-life, and sustain receptor activation, thereby providing an effective pharmacological alternative to standard heterozygous hFSH preparations.

We further evaluated its activity using the ICR mouse strain available in Vietnam. Our results demonstrated that both sc-hFSH LNN and the hFSH NIBSC reference preparation significantly increased ovarian and uterine weights in female mice, with comparable efficacy at equivalent doses. Notably, the single-dose regimen elicited a stronger stimulatory effect compared to the five-dose regimen. This phenomenon can be explained both pharmacokinetically and mechanistically: a single injection maintains hormone levels above the threshold required for sustained FSH receptor (FSHR) activation, thereby promoting continuous signaling in granulosa cells, increasing aromatase expression, estradiol synthesis, and follicular development. In contrast, repeated small doses may fail to achieve sufficient receptor occupancy or may induce partial desensitization, resulting in a reduced overall response. This observation is consistent with previous reports on hyperglycosylated FSH analogs or long-acting FSH variants, which have shown superior *in vivo* efficacy due to longer persistence and more stable receptor binding, even though their *in vitro* activity does not always exceed that of native FSH [13,19–21]. Our earlier study also demonstrated that a single injection of eCG stimulated ovarian growth in immature 21-day-old female mice at lower doses compared with multiple-injection protocols, owing to the long half-life of eCG in circulation [22].

When higher doses of sc-hFSH LNN were assessed, ovarian and uterine responses did not follow a linear dose-dependent pattern. Only the 5 μg/mL dose produced a statistically significant increase in ovarian weight compared with 1 μg/mL, while uterine weight remained largely unchanged. This non-linear response may reflect physiological limitations of the target tissues or concentration-dependent constraints on signaling pathway activity. Indeed, recent studies have demonstrated that different glycoforms of FSH can selectively activate distinct downstream pathways, such as cAMP/PKA versus ERK and β-arrestin, leading to tissue-specific responses [ 23,24]. Glycosylation of FSH thus plays a pivotal role in determining not only the magnitude but also the functional selectivity of signaling cascades that mediate diverse biological effects [24]. However, this hypothesis requires further investigation in other animal models across a broader range of concentrations.

Furthermore, we observed higher serum concentrations of FSH, estradiol, and progesterone in the sc-hFSH LNN and hFSH NIBSC treatment groups compared with the control group at 72 hours after hormone administration at a dose of 5 µg/mL. These findings, together with the observed increase in ovarian weight, suggest that supplementation with exogenous hFSH in immature mice may induce endocrine changes that promote follicular development.

Previous studies have also demonstrated that 21-day-old female mice treated with single-chain hFSH-Fc (which has a different structure from our molecule) exhibited increased ovarian weight compared with solvent-treated controls when evaluated using the Steelman–Pohley assay [18,25]. These authors proposed that FSH binds to specific receptors on ovarian granulosa cells and thereby regulates the selection and development of ovarian follicles, leading to increased ovarian weight. The enhanced activity was suggested to be associated with the prolonged terminal half-life (60–69 hours) of these single-chain molecules in rodent circulation [25].

Our previous research confirmed that sc-hFSH LNN increases intracellular cAMP levels and promotes progesterone synthesis in mLTC/FSHR cells, as well as elevates serum estradiol levels in immature female rats [11]. In another study, a single dose of SAFA-FSH administered to rats resulted in increased ovarian weight and significantly elevated serum estradiol concentrations. This effect was attributed to the long-acting nature of SAFA-FSH, which may upregulate the expression of key steroidogenic enzymes, including aromatase (hCYP19A1) and steroidogenic acute regulatory protein (hSTAR), both of which are critical for estradiol biosynthesis [26].

Similarly, in male mice, sc-hFSH LNN induced weight gain of the seminiferous tubules at all tested doses, whereas total testes weight increased significantly only at the highest dose (5 μg/mL). Serum FSH and testosterone concentrations in immature mice also increased upon treatment with sc-hFSH LNN and hFSH NIBSC. This is consistent with the known biology of FSH in the male gonad, where FSH acts directly on Sertoli cells to promote proliferation, nutrient support for germ cells, and tubular fluid production [15,27–29]. Early Sertoli cell responses, such as fluid accumulation and metabolic changes, can occur rapidly, explaining the broad sensitivity of seminiferous tubule weight. In contrast, changes in total testes mass require more time and higher stimulation to reflect alterations in germ cell proliferation and tissue architecture, as previously noted in mouse and human studies [ 30,31]. Another study reported that the reduction in testicular size and seminiferous tubule area observed in prepubescent YHR mice was associated with decreased FSH levels [32]. The role of FSH in testicular development has been well demonstrated in mouse models carrying mutations in the FSH β-subunit gene and the FSH receptor [ 33,34]. Furthermore, in gonadotropin-deficient hypogonadal mice exhibiting reduced testicular size, sustained FSH activity resulted in increased testicular weight [ 35,36], thereby confirming the direct role of FSH in testicular growth and development.

Reproductive trials in adult females (8 weeks old) showed a higher pregnancy rate in mice treated with sc-hFSH LNN (66.7%) compared with those treated with hFSH NIBSC (55.56%) and controls (33.3%). Although the sample size was limited, this trend underscores the translational relevance of the structural modifications. By covalently linking the α- and β-subunits and incorporating two additional N-glycans in the linker, sc-hFSH LNN likely achieves increased structural stability and prolonged half-life, resulting in more consistent ovarian stimulation and improved oocyte quality. These findings are aligned with prior evidence that single-chain or hyperglycosylated FSH analogs enhance ovulation and pregnancy outcomes in murine models [8,9,37,38]. Notably, mating trials involving both male and female mice treated with sc-hFSH LNN yielded very favorable results. While the pregnancy rate was similar to that of the hFSH NIBSC group, the number of offspring was higher in the sc-hFSH LNN group. This suggests that both sc-hFSH LNN and hFSH NIBSC are highly active in prepubescent and adult mice, with sc-hFSH LNN showing slightly greater biological activity than hFSH NIBSC. However, this conclusion requires confirmation in future studies with larger sample sizes.

Taken together, our findings confirm that the sc-hFSH LNN retains the biological activity of native hFSH while offering distinct pharmacological advantages. The enhanced performance of hFSH LNN in bolus injection regimens, its ability to stimulate both female and male reproductive organs, and the observed trend toward higher pregnancy rates support the hypothesis that structural engineering, specifically covalent subunit linkage and additional N-glycosylation, confers superior *in vivo* efficacy. This work complements and extends previous research on glycoengineered gonadotropins, such as FSH-CTP and other single-chain constructs, reinforcing the concept that optimized glycosylation and structural stability can translate into improved reproductive outcomes [13,21,39–41].

Nevertheless, our study has limitations, including small sample sizes and a short observation window (72 hours), which may not fully capture long-term changes in gonadal histology or steroid hormone dynamics. Future work should include detailed pharmacokinetic profiling of sc-hFSH LNN, glycomics characterization of its glycan structures, and signaling pathway analyses to clarify whether biased FSHR signaling contributes to the observed tissue-specific responses.

## Conclusion

sc-hFSH LNN represents a promising next-generation gonadotropin with the potential to improve fertility treatments. By integrating advances in glycoengineering and single-chain design, this molecule not only retains the efficacy of standard hFSH NIBSC but also demonstrates enhanced stability and biological activity, paving the way for more efficient and sustainable applications in animal reproductive biotechnology.
